# Association Between Psychological Empowerment and Work Engagement Among Rural Nurses: A Latent Profile and Moderation Analysis

**DOI:** 10.1155/jonm/8856627

**Published:** 2026-01-08

**Authors:** Ershan Xu, Huayong Huang, Yanhui Zhou, Wen Tang

**Affiliations:** ^1^ School of Nursing, Hunan University of Medicine, Huaihua, China, hnucm.edu.cn; ^2^ Xiangya School of Nursing, Central South University, Changsha, China, csu.edu.cn; ^3^ Nursing Department, The First Affiliated Hospital, Hengyang Medical, University of South China, Hengyang, China, usc.edu.cn; ^4^ Orthopaedics Center, The First Affiliated Hospital, Hengyang Medical, University of South China, Hengyang, China, usc.edu.cn

**Keywords:** decent work perception, latent profile analysis, psychological empowerment, rural nurse, work engagement

## Abstract

**Aim:**

This study explored the latent classes of psychological empowerment. In addition, we analyzed their relationship with perceptions of decent work and work engagement, thereby providing a scientific basis for enhancing work engagement among rural nurses.

**Background:**

Most studies focus on nurses working in urban areas, with relatively fewer investigations examining those in rural healthcare institutions. While many studies have explored psychological empowerment, decent work perception, and work engagement, few have analyzed the relationships from the perspective of psychological empowerment.

**Methods:**

The sample consisted of clinical nurses from 3 rural healthcare institutions in Hunan Province, China. The nurses’ general information, psychological empowerment, decent work perception, and work engagement scores were assessed using the General Information Scale, Decent Labor Perception Scale, Psychological Empowerment Scale, and Utrecht Work Engagement Scale, respectively. Furthermore, latent profile analysis and moderation analysis were performed.

**Results:**

The total scores of rural nurses’ work engagement were 62.85 (±15.44). Rural nurses’ psychological empowerment exists in three latent categories: low psychological empowerment, competent but constrained, and high psychological empowerment. Level 1 hospitals (OR = 1.95, 95% CI: 1.24–3.09, *p* = 0.004) and internal medicine (OR = 1.18, 95% CI: 1.06–1.31, *p* = 0.004) were associated with low psychological empowerment. Holding a leadership position (OR = 0.28, 95% CI: 0.09–0.88, *p* = 0.029) and days of monthly night shifts (≤ 4) (OR = 0.63, 95% CI: 0.41–0.97, *p* = 0.036) were associated with high psychological empowerment. The category of psychological empowerment has a moderating effect on the relationship between decent work perception and work engagement (*p* < 0.05).

**Conclusions:**

In summary, the levels of work engagement among rural nurses are moderate. Rural nurses’ psychological empowerment is heterogeneous, and attention should be paid to those in the low psychological empowerment group. This study demonstrated that nurses’ psychological empowerment partially moderates the relationship between perceptions of decent work and work engagement. Therefore, emphasizing psychological empowerment and fostering a decent work perception should be considered when exploring measures to promote high work engagement among rural nurses.

**Implications for Nursing Management:**

Based on the study findings, nursing managers should adopt an evidence‐based approach to support psychological empowerment. Particular attention should be given to early identification of nurses at higher risk for low psychological empowerment, especially those in Level 1 hospitals and internal medicine departments. Targeted interventions should also be implemented, such as establishing peer mentoring programs facilitated by highly empowered nurses, creating regular feedback mechanisms, and involving nurses in clinical decision‐making processes. In addition, management should promote leadership opportunities and optimize scheduling to limit night shifts to not more than four per month. Notably, these factors have a significant association with enhanced psychological empowerment.

## 1. Introduction

China’s medical and health system has undergone continuous reform, highlighting the increasing importance of rural healthcare institutions [1]. This new era sets higher requirements for the high‐quality development of rural healthcare, making it an essential component of building a healthy countryside [2]. County‐level and grassroots medical institutions are integral parts of rural healthcare institutions. These typically include county general hospitals, township health centers, community health service centers, and village clinics, which are directly related to the health of the local community [3].

Nurses in these institutions are the most critical human resources. At this step and interface, the economic level in county towns and below is relatively low. In addition, nurses face unique challenges, including limited resources and potentially less access to support systems compared to their urban counterparts in China [4]. Meanwhile, nurses in rural healthcare institutions experience significant work pressure, severe job burnout, a low sense of professional identity, and a strong intention to leave their positions [5]. Consequently, these issues diminish nurses’ work engagement and performance, erode China’s healthcare system, compromise patient outcomes, and ultimately endanger patients’ health [6]. Work engagement refers to the level of dedication, enthusiasm, and commitment an individual demonstrates in their work. It is an essential indicator for measuring employee work attitudes and work efficiency [7]. One study has shown that enhancing work engagement can contribute to increased commitment and improved performance among nurses in healthcare settings [8]. Notably, the level of work engagement among nurses has become a hot topic of research domestically and internationally [9–12]. The existing literature primarily adopts an urban perspective, with empirical studies and theoretical models centered on large medical institutions [13, 14]. However, the distinct realities of rural healthcare, characterized by resource constraints, professional isolation, and a broader scope of practice that requires nurses to be versatile, raise questions about the direct applicability of these findings, which are often urban‐centric. Current research has not adequately clarified how these unique contextual factors specifically influence the work engagement of nurses in rural settings [15–17]. Consequently, the academic community still lacks a contextualized, evidence‐based understanding of how to effectively inspire and sustain work engagement among nurses in rural healthcare institutions. This study aims to fill this evident gap by focusing on nurses working in rural healthcare institutions.

In recent years, the concept of decent work as a perceptual integrative construct that bridges positive psychology and organizational behavior has garnered increasing scholarly attention. Blustein and colleagues define this as employees’ subjective awareness and interpretation of job stability, workplace respect, and occupational safety [18]. Because individuals’ experiences and values differ, the same job may be judged as more or less “decent” across individuals [19]. For example, front‐line employees translate their dignity appraisals into emotional displays that spill over to customers. As a result, when workers themselves feel disrespected, professional identification wanes and service quality deteriorates [20]. To date, the literature on decent work perception has concentrated on two broad streams. The first explores the antecedents of decent work perception, including job autonomy [21], professional identity [22], social support [23], and macrolevel determinants [24, 25]. The second adopts an individual‐level lens to examine behavioral outcomes of decent work perception, including work engagement [26, 27], job embeddedness [28], innovative behavior [29, 30], and dedication [31].

Psychological empowerment is a psychological state that combines four cognitions, meaning, autonomy, competence, and influence, into an integrated perception of active agency at work [32]. By heightening employees’ meaningfulness, allowing them to choose how to perform their tasks freely, and making their actions make a difference, psychological empowerment elicits proactive attitudes and behaviors that help regulate emotions, craft roles, and adapt to situational demands [33], while improving job quality [34]. Moreover, the higher an individual’s psychological empowerment, the greater their work engagement [35, 36].

Furthermore, the self‐determination theory (SDT), formulated by Deci and Ryan [37], posits that sustained and vigorous work motivation stems from the fulfillment of three fundamental psychological needs: autonomy, competence, and relatedness. Notably, four dimensions of psychological empowerment map directly onto SDT needs: meaning (relatedness), autonomy (autonomy), competence (competence), and influence (autonomy and competence). Moreover, the five dimensions of decent work perception, including job rewards, career development, career recognition, work atmosphere, and job position, meet three basic needs of nurses. Thus, they act as an external resource that nourishes nurses’ fundamental psychological needs [38]. Work engagement, defined as a state of vigor, dedication, and absorption, represents the behavioral manifestation of this activated intrinsic motivation [39]. We will frame SDT as the foundational theory that explains why decent work perception (as a supportive context) can foster work engagement (as an outcome) through the satisfaction of basic psychological needs (psychological empowerment).

Meanwhile, current research finds that psychological empowerment does not merely enhance work effectiveness directly. Instead, it serves as a “situational amplifier” and a “resource buffer,” significantly moderating the relationships between other variables. [40, 41]. For example, Cheng et al. [40] found that “When organizational justice is combined with job commitment, if public security police officers perceive a high level of psychological empowerment (such as autonomy in decision‐making, a sense of job control, and influence), they will develop a strong sense of loyalty and conviction, thereby significantly enhancing job satisfaction.” This body of evidence suggests that empowerment not only has a direct effect but also significantly influences how employees perceive and respond to other organizational factors. Therefore, within the theoretical framework of SDT, we hypothesize that psychological empowerment can serve as a moderator between decent work perception and work engagement.

**Figure 1:**
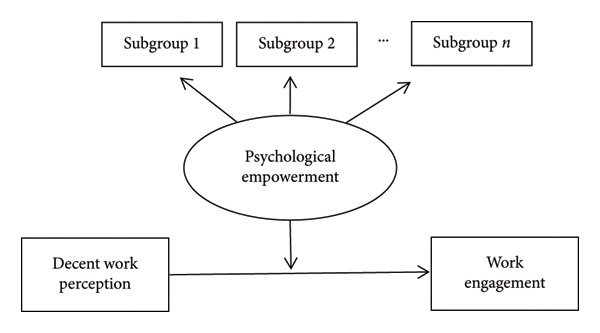
The hypothetical framework for decent work perception, psychological empowerment, and work engagement among rural nurses.

However, psychological empowerment is not a monolithic construct [42]. Individuals cluster into latent classes that differ in empowerment profiles. In addition, the differential impact of these profiles on engagement remains underexamined among the nurses in rural healthcare institutions. Latent profile analysis (LPA) is a valuable tool for gaining a deeper understanding of complex data and making more informed decisions in various fields, including healthcare, education, and social services [43]. This study utilized the latent profiles of psychological empowerment as the independent variable to further investigate its relationship with decent work perception and work engagement. As such, we aimed to provide a scientific basis for enhancing work engagement among nurses in rural healthcare institutions by cultivating psychological empowerment. The hypothesized framework is described in Figure 1.

Hence, the current study was designed to meet the following objectives: (1) Identify latent subgroups with different psychological empowerment types by LPA, (2) compare work engagement among subgroups with LPA‐based psychological empowerment types, and (3) investigate whether profile membership moderates the relationship between decent work perception and work engagement. Crucially, this objective integrates the person‐centered typology (from LPA) into a path analysis framework to test a nuanced research question: Does the effect of decent work perception on engagement depend on a nurse’s overall type of psychological empowerment?

## 2. Methods

### 2.1. Design and Participants

This study conducted a multicenter cross‐sectional survey using a convenience sampling method to select nurses working in rural healthcare institutions (operationally defined as “rural nurses” for this study) in Hunan Province from June to August 2024. Nurses were selected via convenience sampling from one Level 1 hospital, one Level 2 hospital, and one Level 3 hospital. Inclusion criteria were working nurses who completed clinical registration, worked for more than one successive year, and voluntarily participated in this study. Exclusion criteria included intern nurses, nurses who were in the hospital for further training, and nurses who were not in the hospital during the survey period due to sick leave, maternity leave, or retirement. Questionnaires completed in less than 3 minutes or with logical inconsistencies in responses were considered invalid and would not be accepted.

### 2.2. Samples

Based on the standards set by Kendall for estimating sample sizes, it is advised that the sample size be determined as five to ten times the number of items on the scales used [44]. Thus, this research involved the use of a total of 56 items, which included the General Information Questionnaire (13 items), the Decent Work Perception Scale (DWPS) (16 items), the Psychological Empowerment Scale (PES) (12 items), and the Utrecht Work Engagement Scale (WES) (UWES) (15 items). Consequently, the minimum required sample size was calculated as *N* = 10 × (13 + 16 + 12 + 15) = 560. Considering an anticipated rate of invalid questionnaires ranging from 10% to 20%, the final sample size needed was *N* = 560^∗^ (1 + [0.10–0.20]) = 616–674. Ultimately, we collected 848 questionnaires. From these, 625 responses were deemed valid, resulting in an effective response rate of 73.70%.

### 2.3. Data Collection

This study utilized online questionnaires, distributed by the researcher to nurses in the department after obtaining consent from the nursing departments and managers of the three hospitals. The team leader liaised with the hospital’s head of nursing to explain the objectives and procedures of the survey. Following approval and support from hospital management, an anonymous online questionnaire was distributed through WeChat. The link was initially shared with the nursing manager, who then circulated it to nurses working in rural areas during nonworking hours. The purpose and significance of the study were first explained to the nurses, and the questionnaire was distributed anonymously after obtaining their consent. From June to August 2024, 848 questionnaires were distributed, and 625 valid questionnaires were returned.

## 3. Measurements

### 3.1. Demographic Data

This study employed a self‐designed General Information Questionnaire to investigate the sociodemographic characteristics of the hospital level, gender, research team, age, education, year of nursing experience, professional title, marital status, department, number of nights worked monthly, and number of children, as well as employment status, leadership role, and monthly income.

### 3.2. PES

The Chinese version of the PES, developed by Spreitzer [45] and translated and revised by Li et al. [46], was used to assess the level of psychological empowerment among nurses. The scale includes four dimensions of work meaning, competence, autonomy, and influence, with a total of 12 items. Using a 5‐point Likert evaluation method, “1–5” represents the “very much disagree–very much agree,” respectively, increasing level step by step, for a total of 12–60 points. Higher scores indicate higher levels of psychological empowerment among clinical nurses. Exploratory factor analysis revealed that the cumulative variance explained by this scale was 74%. The confirmatory factor analysis results indicate that the PES has acceptable model fit indices, with *χ* ^2^/df = 2.11, RMSEA = 0.042, CFI = 0.990, TLI = 0.987, and SRMR = 0.032. Thus, these indices all meet the acceptable levels. The Cronbach’s *α* coefficient for the PES of rural nurses in this study was 0.774, indicating good reliability and validity of the questionnaire.

### 3.3. DWPS Chinese Version

The scale was developed by Mao et al. [47] and revised by Jiang et al. [48]. The revised questionnaire has good reliability and validity, with a Cronbach’s *α* coefficient of 0.724 and retest reliability of 0.709. The scale includes five dimensions of work: reward decency, work position decency, career development decency, career recognition decency, and work atmosphere decency, with a total of 16 items. The Likert 5‐point scale is used, where scores of “1–5” represent “strongly disagree–strongly agree,” respectively, and the level increases step by step, with a total score ranging from 16 to 80. The higher scores indicate higher levels of decent work perception perceived by clinical nurses. The confirmatory factor analysis suggests that the DWPS has acceptable model fit indices, with *χ* ^2^/df = 4.41, RMSEA = 0.074, CFI = 0.948, TLI = 0.933, and SRMR = 0.045. These indices all meet the acceptable levels. Furthermore, Cronbach’s *α* coefficient for the DWPS among rural nurses in this study was 0.808, indicating good reliability and validity of the questionnaire.

### 3.4. UWES

The UWES scale is the most widely used scale at present, developed by Schaufeli et al. [49]. This scale was first introduced in China by Zhang et al. [50], and its reliability and validity were subsequently tested and analyzed. The scale is divided into three dimensions: concentration, dedication, and vitality, with a total of 15 items. This scale employs a 7‐level Likert scoring method, ranging from “never” (1) to “always” (7). The average score of items below 3 indicates a low level of work engagement, scores between 3 and 5 indicate a medium level of work engagement, and scores above 5 indicate a high level of work engagement. The confirmatory factor analysis results suggest that the WES has acceptable model fit indices, with *χ* ^2^/df = 10.26, RMSEA = 0.122, CFI = 0.886, TLI = 0.862, and SRMR = 0.047. These indices all meet the acceptable levels. Cronbach’s coefficient for the WES among rural nurses in this study was 0.870, indicating good reliability and validity of the questionnaire.

### 3.5. Ethical Considerations

The ethical principles guiding this study, as outlined in the Declaration of Helsinki, informed this investigation, which also obtained authorization from The First Affiliated Hospital of the University of South China (No. 2024LL0715001). All nurses were aware of the purpose and significance of this study before participating, voluntarily agreed to participate, provided signed informed consent, and could withdraw from the study at any time without penalty. The data collected in this study were used solely for data analysis and were not disclosed or shared.

### 3.6. Data Analysis

#### 3.6.1. LPA Analysis and Model Fitting

The LPA was conducted using Mplus 8.3 software to fit the latent profile model of psychological empowerment among rural nurses and to determine the optimal number of classes based on the model fit results. The model evaluation indices included the following: log‐likelihood (LL), Akaike information criterion (AIC), Bayesian information criterion (BIC), adjusted BIC (aBIC), entropy, Lo–Mendell–Rubin adjusted likelihood ratio test (LMRT), and bootstrapped likelihood ratio test (BLRT). Model fit criteria: Lower values of AIC, BIC, and aBIC, along with higher entropy values, indicate a better model fit. More significant *p* values for LMRT and BLRT suggest that a k‐class model is preferable to a k‐1 class model. Based on these criteria, the best‐fitting model was selected, and rural nurses’ psychological empowerment was categorized into different classes. Notably, the basic assumption of LPA is that the probability distribution of responses on observed variables can be explained by a few mutually exclusive latent classes, each with a specific response pattern for the observed variables [51].

#### 3.6.2. Correlation and Descriptive Statistics

Demographic and work engagement characteristics (categorical variables) were described in terms of frequencies and proportions (%). Descriptive statistical analysis was conducted using SPSS 27.0 software, with quantitative data presented as mean ± standard deviation ($\overset{¯}{x}\pm s$). Pearson correlation analysis examined the relationships between psychological empowerment, decent work perception, and work engagement. Comparisons among different psychological empowerment groups were made using one‐way ANOVA, and post hoc comparisons were conducted using LSD *t-*tests. When the variances are not equal (*p* < 0.05), the Kruskal–Wallis test is used.

#### 3.6.3. Moderation Analysis

The moderation analysis of different types of psychological empowerment on the relationship between decent work perception and work engagement was conducted by constructing interaction terms and performing regression analysis. Psychological empowerment and work engagement were first standardized using SPSS 27.0. Dummy variables were created for the different types of psychological empowerment. Interaction terms were calculated between decent work perception and the dummy variables representing psychological empowerment. Regression analysis was then performed using the psychological empowerment dummy variables and the interaction terms as independent variables, with work engagement as the dependent variable.

#### 3.6.4. Normality Test and the Common Method Bias Test

Initially, all variables were tested for normality using histograms and the Q–Q plots. The results indicate that the histograms are approximately bell‐shaped, with higher peaks in the center and lower tails. The data points in the Q–Q plots are closely distributed around the reference line, suggesting that the data follow a normal distribution.

Meanwhile, all variables were tested for standard method bias using Harmon’s single‐factor test through exploratory factor analysis. The unrotated results yielded 20 eigenvalues greater than 1, with the first factor explaining only 38.343% of the variance, below the critical value of 40%. Therefore, standard method bias is not expected to significantly impact this study.

## 4. Results

### 4.1. Demographic Characteristics

A total of 625 nursing interns completed the survey (female, *N* = 620). Complete demographic details are presented in Table 1.

**Table 1:** Demographic characteristics and relevant variable differences in rural nurse work engagement.

Variable	Number	Proportion	*p* value
The level of the hospital			0.329
Level 1 hospital^∗^	26	4.20%	
Level 2 hospital^∗∗^	403	64.50%	
Level 3 hospital^∗∗∗^	196	31.40%	
Gender			0.013
Male	5	0.80%	
Female	620	99.20%	
Age (years)			0.191
≤ 25	37	5.90%	
26∼30	100	16.00%	
31∼35	253	40.50%	
≥ 36	235	37.60%	
Education			0.385
College degree or below	318	50.90%	
Bachelor degree	306	49.00%	
Graduate student and above	1	0.10%	
Years of nursing experience			0.028
≤ 5	49	7.80%	
6∼15	399	63.80%	
16∼25	112	17.90%	
> 25	65	10.40%	
Department			0.358
Internal medicine	190	30.40%	
Surgery	167	26.70%	
Intensive care unit	23	3.70%	
Operating room	19	3.00%	
Emergency	23	3.70%	
Others	203	32.50%	
Professional title			0.276
Staff nurse	43	6.90%	
Senior nurse	160	25.60%	
Supervisor nurse	389	62.20%	
Co‐chief nurse	30	4.80%	
Chief nurse	3	0.50%	
Marital status			0.001
Married	541	86.60%	
Unmarried	67	10.70%	
Divorce	16	2.60%	
Widowed	1	0.20%	
Number of children you have			0.342
0	83	13.30%	
1∼2	504	80.60%	
> 2	38	6.10%	
Employment status			0.059
Formal	188	30.10%	
Contract	437	69.90%	
Whether to hold a leadership position			0.000
Yes	47	7.50%	
No	578	92.50%	
Days of the night shift monthly			0.616
≤ 4	458	73.30%	
5∼7	147	23.50%	
≥ 8	20	3.20%	
Monthly income (CNY)			0.452
≤ 3000	100	16.00%	
3001∼6000	483	77.30%	
6001∼9000	38	6.10%	
> 9000	4	0.60%	

^∗^Level 1: Primary care institutions (< 100 beds) focused on preventive care and common illnesses.

^∗∗^Level 2: District‐level hospitals (101–500 beds) that handle complex cases and regional referrals.

^∗∗∗^Level 3: Regional medical centers (> 500 beds) that provide comprehensive/specialized care, teaching, and research.

### 4.2. Correlational Analysis of Psychological Empowerment, Decent Work Perception, and Work Engagement

The total scores of psychological empowerment, decent work perception, and work engagement of nurses working in rural healthcare institutions were (39.85 ± 7.73), (49.42 ± 11.11), and (62.85 ± 15.44), respectively. In addition, the mean scores of total entries were (3.32 ± 0.64), (3.09 ± 0.69), and (4.19 ± 1.03), respectively. The average scores for each dimension of work engagement were vigor (4.26 ± 1.08), dedication (4.35 ± 1.11), and absorption (3.97 ± 1.06). The results of Pearson correlation analysis showed that the total score and the scores on each dimension of psychological empowerment, work engagement, and decent work perception were positively correlated (both *p* < 0.01) (Table S1).

### 4.3. LPA of Psychological Empowerment

**Figure 2:**
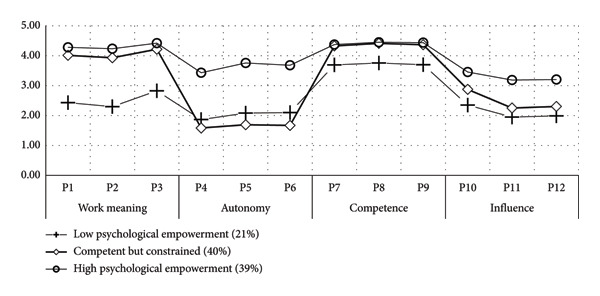
Varying profiles of psychological empowerment among rural nurses.

Based on the sequence of type numbers, psychological empowerment was divided into five type models, starting with a two‐type model and sequentially estimating each model. As shown in Table 2, the values of AIC, BIC, and aBIC gradually decreased with the increase in the number of types. Notably, the most significant drops occurred between the two‐type and three‐type models. The entropy values were all greater than 0.8. The LMRT and BLRT for the two‐type and three‐type models were both significant (*p* < 0.05). This indicates that the optimal fit was achieved when the psychological empowerment type model for nurses working in rural healthcare institutions was divided into a three‐type model. At this point, the type probabilities for the three‐type model were 21%, 40%, and 39%, respectively. Based on LPA and in conjunction with the theory of psychological empowerment, the latent types of psychological empowerment were named. The score distribution of the latent types of psychological empowerment on the 12 items (P1∼P12) of the PES is shown in Figure 2. Type 1, characterized by universally low scores on the four dimensions of psychological empowerment, accounted for 21% (130/625) of the total number of participants and was named the low psychological empowerment group. Type 2 competence and work meaning dimensions of psychological empowerment are relatively high, with autonomy and influence dimensions having the lowest scores, accounting for 40% (251/625) of the total number of participants. Moreover, rural nurses identify with their work content and believe they are competent to perform it; yet, they are constrained in decision‐making participation, resource control, and work impact. Therefore, it was named the competent but constrained group. Type 3 had the highest scores on all items, accounting for 39% (244/625) of the total number of participants, and was named the high psychological empowerment group. The average probability of attribution for the three groups ranged from 94.3% to 95.8% (Table 3), indicating that the LPA classification results in this study are credible. The differences in total scores and scores on each dimension of the PES among the three groups were statistically significant (*p* < 0.01 for all comparisons). Importantly, the high psychological empowerment group had the highest total scores on each dimension, as shown in Table 4.

**Table 2:** LPA of psychological empowerment and model fit results (*N* = 625).

Profile	AIC	BIC	aBIC	LMRT (*p* value)	BLRT (*p* value)	Entropy	Group size for each profile (ratio)				
							1	2	3	4	5
1	21059.351	21165.857	21089.660								
2	19638.943	19803.140	19685.670	0.0055	0.0000	0.872	357 (0.57)	268 (0.43)			
3	18812.532	19034.420	18875.677	0.0001	0.0000	0.887	130 (0.21)	251 (0.40)	244 (0.39)		
4	18383.740	18663.318	18463.302	0.1296	0.0000	0.874	231 (0.37)	183 (0.29)	107 (0.17)	104 (0.17)	
5	17934.770	18272.039	18030.749	0.1937	0.0000	0.898	113 (0.18)	13 (0.02)	178 (0.28)	205 (0.33)	116 (0.19)

**Table 3:** Average probability of attribution for each potential profile.

Class	Profile 1 (%)	Profile 2 (%)	Profile 3 (%)
1	0.943	0.041	0.016
2	0.027	0.946	0.028
3	0.004	0.037	0.958

**Table 4:** Total psychological empowerment score and each dimension score of the three potential categories (score, $\overset{¯}{x}\pm s$).

Category	The total score	Work meaning	Autonomy	Competence	Influence
Low psychological empowerment	2.58 ± 0.46	2.51 ± 0.66	2.03 ± 0.87	3.70 ± 0.83	2.05 ± 0.78
Competent but constrained	3.13 ± 0.32^∗^	4.06 ± 0.53^∗^	1.62 ± 0.58^∗^	4.37 ± 0.52^∗^	2.47 ± 0.92^∗^
High psychological empowerment	3.91 ± 0.42^#^	4.31 ± 0.54^#^	3.63 ± 0.68^#^	4.42 ± 0.49^#^	3.28 ± 0.82^#^
F/P	535.59/< 0.001	465.86/< 0.001	560.87/< 0.001	72.16/< 0.001	97.51/< 0.001

*Note:* Compared to the low psychological empowerment group, ^∗^ *p* < 0.01; compared to the medium psychological empowerment group, ^#^ *p* < 0.01.

### 4.4. LPA‐Based Differences in UWES Scores

The three psychological empowerment groups showed statistically significant differences in levels of work engagement and their dimensions. Compared to the high psychological empowerment group, the low psychological empowerment group demonstrated significantly lower levels of overall work engagement, as well as each of its constituent dimensions. All *p* values were less than 0.001 (see Table 5).

**Table 5:** Work engagement comparison in three potential types of psychological empowerment groups (score, $\overset{¯}{x}$ ± *s*).

Variable	Low psychological empowerment	Competent but constrained	High psychological empowerment	*F*/*p*
Total work engagement score	3.31 ± 0.85	4.17 ± 0.87	4.69 ± 0.95	100.31/< 0.001
Vigor	3.37 ± 0.92	4.24 ± 0.92	4.77 ± 1.00	91.79/< 0.001
Dedication	3.43 ± 0.93	4.33 ± 0.96	4.87 ± 1.02	93.14/< 0.001
Absorption	3.14 ± 0.91	3.95 ± 0.93	4.44 ± 0.98	80.88/< 0.001

### 4.5. Univariate and Multivariate Logistic Regression Results for Predicting External Features on the 3‐Class Pattern of Psychological Empowerment

Level 1 hospitals (OR = 1.95, 95% CI: 1.24–3.09, *p* = 0.004) and internal medicine (OR = 1.18, 95% CI: 1.06–1.31, *p* = 0.004) were associated with low psychological empowerment. In addition, those who hold a leadership position (OR = 0.28, 95% CI: 0.09–0.88, *p* = 0.029) and days of night shift monthly (≤ 4) (OR = 0.63, 95% CI: 0.41–0.97, *p* = 0.036) were prone to high psychological empowerment (see Table 6).

**Table 6:** Univariate and multivariate logistic regression results for predicting external features on the 3‐class pattern.

Variables	Univariate analysis		Multivariate analysis			
	*X* ^2^	*p*	Class 2 vs. Class 1 OR (95% CI)	*p*	Class 3 vs. Class 1 OR (95% CI)	*p*
The level of the hospital (Level 1 hospital as ref)	12.43	0.014	1.95 (1.24–3.09)	**0.004**	1.19 (0.75–1.87)	0.462
Gender (male as ref)	1.41	0.558				
Age (years) (≤ 25 as ref)	25.05	0.000	1.44 (0.92–2.24)	0.110	0.92 (0.59–1.42)	0.659
Education (college degree or below as ref)	2.65	0.620				
Years of nursing experience (≤ 5 as ref)	21.47	0.002	1.01 (0.60–1.69)	0.972	0.82 (0.48–1.38)	0.450
Department (internal medicine as ref)	18.89	0.042	1.09 (0.98–1.22)	0.114	1.18 (1.06–1.31)	**0.004**
Professional title (staff nurse as ref)	28.63	0.000	1.07 (0.67–1.68)	0.788	0.98 (0.63–1.55)	0.939
Marital status (married as ref)	8.77	0.187				
Number of children you have (0 as ref)	13.32	0.010	1.31 (0.71–2.39)	0.384	1.04 (0.57–1.89)	0.905
Employment status (formal as ref)	7.59	0.022	0.72 (0.37–1.39)	0.335	0.57 (0.29–1.08)	0.085
Whether to hold a leadership position (yes as ref)	18.23	0.000	1.177 (0.34–4.09)	0.797	0.28 (0.09–0.88)	**0.029**
Days of the night shift monthly (≤ 4 as ref)	13.11	0.010	0.76 (0.49–1.16)	0.208	0.63 (0.41–0.97)	**0.036**
Monthly income (CNY) (≤ 3000 as ref)	19.28	0.002	0.93 (0.52–1.63)	0.787	1.28 (0.70–2.14)	0.471

*Note:* The bold values indicate that the corresponding statistical tests yielded *p* values less than 0.05, demonstrating statistical significance.

### 4.6. The Moderating Role of Psychological Empowerment Types Between Decent Work Perception and Work Engagement

The moderating effect of the three psychological empowerment groups on the relationship between decent work perception and work engagement was analyzed using the method of constructing interaction terms and regression analysis. Compared to the low psychological empowerment group, the interaction terms between decent work perception and the competent but constrained group, as well as the high psychological empowerment group, had a significant impact on work engagement, with *R* ^2^ = 0.435 and all *p* values less than 0.01. This suggests that the psychological empowerment categories have a moderating effect on the relationship between perceptions of decent work and work engagement (Table 7).

**Table 7:** The moderating effect of different categories of psychological empowerment on the relationship between decent work and work engagement.

Independent variable	Unstandardized coefficients		t	*p*	Collinearity statistics	
	B	Standard error			Tolerance	VIF
Constant	−0.857	0.066	−12.955	0.000		
Competent but constrained	0.904	0.082	11.011	0.000	0.562	1.781
High psychological empowerment	0.477	0.044	10.925	0.000	0.501	1.996
Perception of decent work × competent but constrained	0.391	0.055	7.093	0.000	0.974	1.027
Perception of decent work × high psychological empowerment	0.337	0.027	12.616	0.000	0.805	1.242

*Note:* Dependent variable: work engagement.

## 5. Discussion

The work engagement score was 4.19 (±1.03) points, at a moderate level, with a relatively lower score in the absorption dimension. This may be due to the heavy workload of nursing tasks, coupled with the fact that 99.20% of the participants in this study are female, 78.1% are over 30 years old, 86.60% are married, and 86.70% have more than one child. The female demographic bears a greater share of family responsibilities, making it challenging to balance work and family life effectively, which in turn reduces their ability to engage fully in their work. Therefore, hospitals should offer flexible scheduling, encourage time off, and provide resources to help nurses manage stress and balance their personal and professional lives.

This study was the first to examine latent profiles of psychological empowerment using LPA in a sample of nurses working in rural healthcare institutions. Using the LPA, individuals can be categorized into groups that share similarities but differ from individuals in other groups [52]. Through the LPA of psychological empowerment, rural nurses’ psychological empowerment can be categorized into three latent groups: low psychological empowerment, competent but constrained, and high psychological empowerment, with each group accounting for 21%, 40%, and 39%, respectively. These results confirm the heterogeneity of psychological empowerment. In addition, an ANOVA of work engagement among rural nurses with different levels of psychological empowerment revealed statistically significant differences in work engagement and its dimensions across various categories of psychological empowerment. We observed a significant difference in work engagement among three LPA‐based psychological empowerment subgroups. This study aligns with previous research, which suggests that psychological empowerment can have a direct, positive influence on work engagement [53]. Furthermore, the underlying mechanism may be that psychological empowerment enhances nurses’ self‐efficacy, enabling them to proactively address challenges rather than responding passively [54].

Nurses with higher levels of psychological empowerment are more likely to recognize their professional worth, thereby enhancing their sense of well‐being and reducing job burnout [55]. Two studies have highlighted that a high level of empowerment is associated with lower turnover, reduced stress, and increased workplace satisfaction and commitment, leading to positive health outcomes [56, 57]. Individuals in the “low psychological empowerment” group (21%), due to a lack of sense of meaning, competence, and influence, are more susceptible to issues such as job burnout and increased turnover intention when facing the challenges of rural healthcare work. Therefore, these individuals should be the primary target for management interventions.

Nursing managers should accurately identify nurses in the low psychological empowerment group and implement targeted interventions to address their needs. Simultaneously, they should leverage the exemplary role of nurses in the high psychological empowerment group to foster mentorship and peer support. By optimizing work design, providing opportunities for participation in decision‐making, and strengthening positive feedback, managers can comprehensively enhance nurses’ sense of meaning, self‐determination, and influence. This approach will also facilitate the transition of nurses from a state of “low” or “medium” to one of “high,” ultimately improving overall work engagement levels.

This study finds that psychological empowerment significantly moderates the relationship between decent work perception and work engagement. These results suggest that enhancing psychological empowerment can amplify the positive impact of decent work perception on work engagement, ultimately elevating the quality of nursing care in rural settings. These moderating effects can also be explained through the lens of SDT [58]. Decent work perception, comprising factors such as fair rewards, career opportunities, and a supportive atmosphere, functions as a foundational contextual resource that satisfies nurses’ basic psychological needs for autonomy, competence, and relatedness [59]. This satisfaction is intrinsically manifested as a state of psychological empowerment, where nurses experience their work as meaningful, feel competent in their abilities, and have a sense of self‐determination and impact [45]. Crucially, this resource‐rich state of empowerment does not merely translate directly into engagement; it also amplifies the very process of resource utilization. When nurses feel psychologically empowered, they are more likely to proactively leverage the resources provided by a decent work environment. For instance, a nurse who feels empowered (high competence and autonomy) is better equipped to capitalize on professional development opportunities. As such, they derive greater competence, satisfaction, and, consequently, deeper work engagement. Conversely, in the absence of empowerment, substantial contextual resources may not be fully utilized or translated into sustained motivation. Therefore, psychological empowerment acts as a “situational amplifier” within the SDT framework. Moreover, it intensifies the positive cycle wherein contextual resources (decent work) fulfill psychological needs, fueling the motivated state of work engagement. Thus, these findings underscore that empowering nurses is not only beneficial in itself but also pivotal for maximizing the motivational return on investments made in creating decent working conditions.

As shown in Table 7, although the “competent but constrained” profile reported a lower level of psychological empowerment than the “high empowerment” profile, its interaction with decent work perception was stronger (*B* = 0.391) than that of the “high psychological empowerment” profile (*B* = 0.337). This apparent paradox can be interpreted as rural nurses who lack autonomy being more sensitive to the concept of decent work. The characteristics of the competent but constrained group are high competence but low autonomy. Thus, their psychological needs are met in an unbalanced way. For this group, the most core role of decent work perception is that it immensely satisfies their missing “autonomy” needs. Therefore, these individuals are more sensitive to the decent work perception. In contrast, the high psychological empowerment group already possesses a wealth of personal resources. Thus, the incremental value of decent work is subject to diminishing returns, resulting in a flatter slope of the relationship between decent work and its benefits.

## 6. Conclusion

In summary, the levels of work engagement among rural nurses are moderate. Rural nurses’ psychological empowerment is heterogeneous, and attention should be paid to those in the low psychological empowerment group. This study showed that nurses’ psychological empowerment subtypes partially moderate the relationship between decent work perception and work engagement. Thus, emphasizing psychological empowerment and fostering a decent work perception should be considered when exploring measures to promote high work engagement among rural nurses.

## 7. Implications for Nursing Management

This study demonstrates that psychological empowerment plays a critical role in enhancing work engagement and decent work perception among rural nurses. To translate these findings into actionable strategies, the following concrete recommendations are proposed for nursing managers, hospital administrators, and policymakers.

For policymakers: Integrating psychological empowerment as a key indicator in the evaluation system for rural healthcare institutions and developing specific funding programs to support empowerment training, mental health services, and leadership development within the National Nursing Development Plan (2021–2025); establishing policies that standardize employment conditions, thereby ensuring contractual nurses receive equal benefits, pay scales, and career advancement opportunities as permanent staff.

For hospital administrators: Implementing an empowerment‐based management model that includes clear delegation of authority, individualized job design, and participatory decision‐making processes to enhance autonomy and control among nurses working in rural healthcare institutions; developing structured career progression pathways and offering regular competency‐based training, particularly for the 50.9% of nurses with an associate degree or lower, to improve confidence and perceived empowerment.

For nursing managers: Adopting an evidence‐based approach to support psychological empowerment. Particular attention should be given to early identification of nurses at higher risk for low psychological empowerment, especially those working in Level 1 hospitals and internal medicine departments. Targeted interventions should also be implemented, such as establishing peer mentoring programs facilitated by highly empowered nurses, creating regular feedback mechanisms, and involving nurses in clinical decision‐making processes. In addition, management should promote leadership opportunities and optimize scheduling to limit night shifts to not more than four per month, as these factors have been demonstrated to have a significant association with enhanced psychological empowerment.

By adopting these multilevel strategies, healthcare institutions can significantly enhance psychological empowerment among rural nurses, leading to improved job satisfaction, work engagement, and ultimately, higher quality of patient care in underserved areas.

## 8. Limitations

This study has several limitations. First, the sample was drawn exclusively from three rural healthcare institutions in Hunan Province, which limits the generalizability of the findings and introduces selection bias. Future studies should thus validate these results in more diverse rural healthcare institutions. Second, the cross‐sectional design prevents the determination of causality; longitudinal research is needed to confirm the observed relationships.

## Conflicts of Interest

The authors declare no conflicts of interest.

## Author Contributions

Study design: Ershan Xu, Huayong Huang, and Yanhui Zhou. Data collection: Wen Tang and Huayong Huang. Data analysis: Ershan Xu and Huayong Huang. Study supervision: Huayong Huang and Yanhui Zhou. Manuscript writing: Ershan Xu. Critical revisions for important intellectual content: Huayong Huang. Article revision: Huayong Huang.

## Funding

This work was supported by the Natural Science Foundation of Hunan Province (2024JJ6408), the Hunan Nursing Association (HNKYP202411), and the Scientific Research Project of the Hunan Provincial Department of Education (23B1044).

## Supporting Information

Table S1: Pearson correlation analysis among the variables of psychological empowerment, decent work perception, and work engagement.

## Supporting information


**Supporting Information** Additional supporting information can be found online in the Supporting Information section.

## Data Availability

The data that support the findings of this study are available from the corresponding author upon reasonable request.
