# Correction to “Association Between Psychological Empowerment and Work Engagement Among Rural Nurses: A Latent Profile and Moderation Analysis”

**DOI:** 10.1155/jonm/9806803

**Published:** 2026-06-18

E. Xu, H. Huang, Y. Zhou, W. Tang, “Association Between Psychological Empowerment and Work Engagement Among Rural Nurses: A Latent Profile and Moderation Analysis,” *Journal of Nursing Management*, 2026, 8856627, https://doi.org/10.1155/jonm/8856627.

In the article, there are errors in the referencing. In the list of references, reference [59] should be renumbered as [54], and references [54]–[58] should be renumbered as [55]–[59]. The citation numbers in the article body remain unchanged.

We apologize for these errors.

