# Supplementary material for: Association Between Psychological Empowerment and Work Engagement Among Rural Nurses: A Latent Profile and Moderation Analysis
Source: J Nurs Manag. 2026 Jan 8;2026:8856627. doi: 10.1155/jonm/8856627 (PMC12783919; doi:10.1155/jonm/8856627)
Supplement: Supplementary file 1 — Supporting Information Additional supporting information can be found online in the Supporting Information section. [file JONM-2026-8856627-s001.docx]

| Table S1. Pearson correlation analysis among the variables of psychological empowerment, perception of decent work, and work engagement (r). | | | | | | | | | | | | | | | |
| --- | --- | --- | --- | --- | --- | --- | --- | --- | --- | --- | --- | --- | --- | --- | --- |
| Variables | **Total psychological**  **empowerment score** | Work meaning | Autonomy | Competence | Influence | **Total**  **decent work**  **perception**  **score** | Job rewards | Job position | Career development | Career recognition | Work atmosphere | **Total work**  **engagement score** | Vigor | Dedication | Absorption |
| **Total psychological empowerment score** | | | | |  | .623^**^ |  |  |  |  |  | .591^**^ |  |  |  |
| Work meaning |  | 1 | .309^**^ | .459^**^ | .324^**^ |  | .447^**^ | .399^**^ | .452^**^ | .473^**^ | .450^**^ |  | .526^**^ | .570^**^ | .481^**^ |
| Autonomy |  | .309^**^ | 1 | .109^**^ | .447^**^ |  | .419^**^ | .322^**^ | .449^**^ | .417^**^ | .371^**^ |  | .383^**^ | .391^**^ | .357^**^ |
| Competence |  | .459^**^ | .109^**^ | 1 | .205^**^ |  | .156^**^ | .213^**^ | .232^**^ | .233^**^ | .319^**^ |  | .390^**^ | .362^**^ | .287^**^ |
| Influence |  | .324^**^ | .447^**^ | .205^**^ | 1 |  | .347^**^ | .226^**^ | .324^**^ | .372^**^ | .275^**^ |  | .326^**^ | .335^**^ | .340^**^ |
| **Total decent work perception score** | | | | | | 1 |  |  |  |  |  | .643^**^ |  |  |  |
| Job rewards |  | .447^**^ | .419^**^ | .156^**^ | .347^**^ |  | 1 | .552^**^ | .590^**^ | .642^**^ | .459^**^ |  | .485^**^ | .504^**^ | .483^**^ |
| Job position |  | .399^**^ | .322^**^ | .213^**^ | .226^**^ |  | .552^**^ | 1 | .581^**^ | .528^**^ | .527^**^ |  | .417^**^ | .434^**^ | .390^**^ |
| Career development |  | .452^**^ | .449^**^ | .232^**^ | .324^**^ |  | .590^**^ | .581^**^ | 1 | .626^**^ | .679^**^ |  | .505^**^ | .554^**^ | .492^**^ |
| Career recognition |  | .473^**^ | .417^**^ | .233^**^ | .372^**^ |  | .642^**^ | .528^**^ | .626^**^ | 1 | .586^**^ |  | .525^**^ | .594^**^ | .527^**^ |
| Work atmosphere |  | .450^**^ | .371^**^ | .319^**^ | .275^**^ |  | .459^**^ | .527^**^ | .679^**^ | .586^**^ | 1 |  | .539^**^ | .591^**^ | .473^**^ |
| **Total work engagement score** | | | | | | |  |  |  |  |  | 1 |  |  |  |
| Vigor |  | .526^**^ | .383^**^ | .390^**^ | .326^**^ |  | .485^**^ | .417^**^ | .505^**^ | .525^**^ | .539^**^ |  | 1 | .873^**^ | .867^**^ |
| Dedication |  | .570^**^ | .391^**^ | .362^**^ | .335^**^ |  | .504^**^ | .434^**^ | .554^**^ | .594^**^ | .591^**^ |  | .873^**^ | 1 | .834^**^ |
| Absorption |  | .481^**^ | .357^**^ | .287^**^ | .340^**^ |  | .483^**^ | .390^**^ | .492^**^ | .527^**^ | .473^**^ |  | .867^**^ | .834^**^ | 1 |
